# Spatial, Phylogenetic, Environmental and Biological Components of Variation in Extinction Risk: A Case Study Using *Banksia*

**DOI:** 10.1371/journal.pone.0154431

**Published:** 2016-05-05

**Authors:** Marcel Cardillo, Alexander Skeels

**Affiliations:** Macroevolution & Macroecology Group, Research School of Biology, Australian National University, Canberra, 0200, Australia; University of Colorado, UNITED STATES

## Abstract

Comparative analyses of extinction risk routinely apply methods that account for phylogenetic non-independence, but few analyses of extinction risk have addressed the possibility of spatial non-independence. We explored patterns of extinction risk in *Banksia*, a plant genus largely endemic to Australia’s southwest biodiversity hotspot, using methods to partition the variance in two response variables (threat status and range size) into phylogenetic, spatial, and independent components. We then estimated the effects of a number of biological and external predictors on extinction risk independently of phylogeny and space. The models explained up to 34.2% of the variation in range size and up to 9.7% of the variation in threat status, nearly all of which was accounted for by the predictors, not by phylogeny or space. In the case of *Banksia*, therefore, high extinction risk can be clearly linked with biological syndromes (such as a brief flowering period) or geographic indicators of human impact (such as extensive habitat loss), but cannot be predicted from phylogenetic relatedness or geographic proximity.

## Introduction

Over the past few decades, numerous studies have used a comparative approach in an attempt to identify particular biological syndromes or ecological strategies that effectively distinguish extinction-prone from non extinction-prone plant species. It has been suggested that extinction-prone species should be characterized by specialized ecological requirements (e.g.[1]), small or highly variable population sizes (e.g. [2]), or by a life-history strategy that favours individual persistence over high fecundity (e.g.[3,4]). While many studies have found support for such predictions, biological attributes usually explain very little of the variation in extinction risk across plant species. Furthermore, their effects appear to be highly variable and context-dependent [1,5–8], and may interact in complex ways with external factors [9]. In general, variation in extinction risk among plant species seems to be associated with varying degrees of exposure to external threatening processes (e.g. habitat loss or overexploitation), mediated by species’ intrinsic biological traits, and by the environmental context in which species live. Hence, any comparative analysis of plant extinction risk should consider all three of these elements [10].

In addition, it has become standard practice for comparative analyses of extinction risk to use methods that address the potentially confounding effects of phylogenetic non-independence [11]. This is important because the intrinsic species traits that may mediate the effects of threatening processes on extinction risk are usually expected to show phylogenetic signal [12]. On the other hand, analyses of extinction risk that address the issue of spatial non-independence are far less common (but see[13,14]). Nonetheless, spatial non-independence could be at least as important as phylogenetic non-independence, because (1) many of the environmental variables that characterize a species’ distribution are likely to be spatially autocorrelated, and (2) many of the threatening processes themselves may be spatially autocorrelated. This means that the residuals of a model of extinction risk that includes geographic variables may suffer from (spatial) non-independence, violating a fundamental statistical assumption, unless this non-independence is accounted for by the model. Furthermore, phylogenetic and spatial autocorrelation may themselves be correlated because the historic legacy of speciation leads to phylogenetic conservatism in the location of species distributions [15].

As well as the need to account for the possible effects of phylogenetic and spatial autocorrelation in comparative analyses, it may also be instructive to quantify their effects and compare the relative contribution of spatial and phylogenetic autocorrelation, intrinsic traits, environmental features, and threatening processes, on the variation in extinction risk among species. This would allow us to expand on the question of whether certain biological traits predict extinction risk, to ask a more general question: if two species have a similar level of extinction risk, is it because (1) they have similar biological traits; (2) they inhabit similar environments; (3) they are exposed to the same threatening processes; (4) they are closely related; or (5) they occur in the same region?

In this study, we explore this way of partitioning variation in extinction risk in the plant genus *Banksia* L.f. Of the 170 species of *Banksia*, 155 (91%) are endemic to Australia’s Southwest Botanical Province (SWBP), a global biodiversity hotspot. All *Banksia* are woody shrubs or trees, but their growth forms vary greatly, from prostrate ground covers to trees of >10m. The primary threat to *Banksia* species is habitat loss: much of the SWBP has suffered very high rates of native vegetation clearance and fragmentation, and the ranges of many species have contracted, in some cases severely [6]. *Banksia* are also exposed to a variety of other threatening processes, including altered fire regimes, harvesting for the flower industry, and diseases such as the fungal pathogen *Phytophthora cinnamomi* [6,16].

To quantify extinction risk for each species, we use two measures, threat status and geographic range size. In many previous comparative studies of extinction risk, range size has been treated as a predictor of threat status rather than an alternative response variable (e.g.[17,18]). However, range size forms part of the criteria by which Australian species are assigned to threat status categories (the same as those used for the IUCN Red List), leading to possible circularity [19]. We therefore examine range size as a response variable, assuming that species with smaller ranges are likely to be more vulnerable to extinction. The two responses are not independent, therefore, but threat status captures some aspects of extinction risk, such as population size, fragmentation or decline rate, not captured by range size.

## Materials and Methods

### Compilation of datasets

The *Banksia* phylogeny we used as the framework for our comparative tests is from [20], which includes 158 of the 170 known species of *Banksia*. Replicated analyses using a sample of trees from the Bayesian posterior distribution produced very little variation in the results, so for simplicity we present results based only on the maximum clade credibility tree.

To quantify extinction risk, each species was assigned a value based on its conservation status under the Environmental Protection and Biodiversity Conservation Act (EPBC), which categorizes extant threatened species as vulnerable, endangered, or critically endangered. For species not considered threatened under the EPBC, we applied a further categorization using the Priority Levels of the Western Australian Declared Rare and Priority Conservation List (DRPLC), which covers >90% of *Banksia* species. Priority 1 includes species of which less than five populations are known and all are under immediate threat; Priority 2 includes species with fewer than five populations, some but not all of which are under immediate threat; Priority 3 includes species of which several populations are known and may be considered rare; Priority 4 includes species known to be rare but are not considered threatened. Species not listed as threatened under the EPBC or listed as a priority Species under the DRPLC were considered “least concern”. We converted the extinction risk categories into an ordinal variable as follows: 0 = least concern, 1 = DRPLC Priority three and four, 2 = DRPLC Priority two, 3 = DRPLC Priority one, 4 = EPBC Vulnerable, 5 = EPBC Endangered, 6 = EPBC Critically Endangered.

Our choice of variables to include as predictors in our models was guided by previous work on *Banksia* and other plant taxa, but was limited by data availability and coverage. Species-level values of four biological traits (mean seed number, mean adult height, number of months in flower per year, and fire response strategy) were compiled from flora entries and monographs [21–23]. The preferred habitat structure for each species was summarized from information presented in [21]and [24], and coded as a set of ordered categories of increasing structural complexity: 1 = shrubland; 2 = shrubland/woodland; 3 = woodland; 4 = woodland/open forest; 5 = closed forest.

In addition to data from species-specific descriptions, we used species distributions to calculate summary values of spatial environmental layers for each species. To estimate species distributions we used occurrence records from all herbaria across Australia, available from the Australian Virtual Herbarium (http://avh.chah.org.au). After cleaning the set of records by removing duplicates and obvious outliers (e.g. those well outside a species distribution limits indicated in [22] and [23], we uploaded the records into the Atlas of Living Australia (http://www.ala.org.au), and converted the points to a grid format with a resolution of 100km^2^. For each grid cell we then extracted a value for mean annual precipitation, mean annual temperature, soil depth, soil pH, and % of natural vegetation cover remaining. For each of these variables, we obtained a mean value across the grid cells occupied by each species. We did not include measures of environmental niche breadth, because such measures are frequently biased by sampling effects [25], and hence confounded with species abundance and range size, one of our response variables. We did not attempt to include all axes of environmental variation that might limit the distribution of *Banksia* species, for example measures of climatic extremes such as maximum temperature in the driest month. Our aim was to capture a small set of the most general environmental variables that are likely to have broad influence across the genus, rather than variables that may only be important for a small number of species in particular regions. The dataset and geographic centroids are provided in S1 Table and S2 Table.

### Analyses

Freckleton & Jetz [26] presented a generalized least-squares model that partitions the variance in a response variable into a linear combination of phylogenetic, spatial, and independent (neither phylogenetic nor spatial) variance components:

V(ϕ,λ)=γh+λ′Σ+ϕW

Where *Σ* is a variance-covariance matrix describing the phylogenetic distances among species, ***W*** is a variance-covariance matrix describing spatial distances among species, and ***h*** is a vector of tip heights from the phylogeny. The parameter *λ*′ = (1 − *ϕ*)*λ*, where *λ* is Pagel’s measure of phylogenetic signal [27], and represents the phylogenetic component. The parameter *ϕ* represents the spatial component, and *γ* = (1 − *ϕ*)(1 − *λ*) represents the independent component. The three parameters sum to one, and can be interpreted as the relative contribution of phylogenetic, spatial and independent effects, provided the phylogenetic and spatial matrices are scaled to the same units.

We generated a phylogenetic matrix from the *Banksia* maximum clade credibility tree using the function “vcv.phylo” in the ape library for R. To generate the spatial matrix we used the function “earth.dist” in the fossil library for R to obtain great-circle distances between the centroids of species distributions. We used the function “regress” in the regress library for R [28] to fit models in which the phylogenetic and spatial variance components are jointly estimated by maximum likelihood, and we then used these to calculate *ϕ*, *λ’*, and *γ*.

To fit models we used a two-stage approach. For each response variable (threat status and range size), we first fitted a full model that included all predictor variables together with phylogenetic and spatial matrices, and simplified this to a minimum adequate model in which all predictors were significant. This identified the sets of factors associated with extinction risk independently of one another, and independently of phylogenetic and spatial effects. We then took these sets of predictors forward for further model comparison. We used the Akaike Information Criterion (AIC) to compare the fit of the full models (predictors + phylogeny + space) to models including predictors only, phylogeny + space only, predictors + phylogeny, and predictors + space. The R code used for the analysis is provided in S1 Text.

## Results

**Fig 1:**
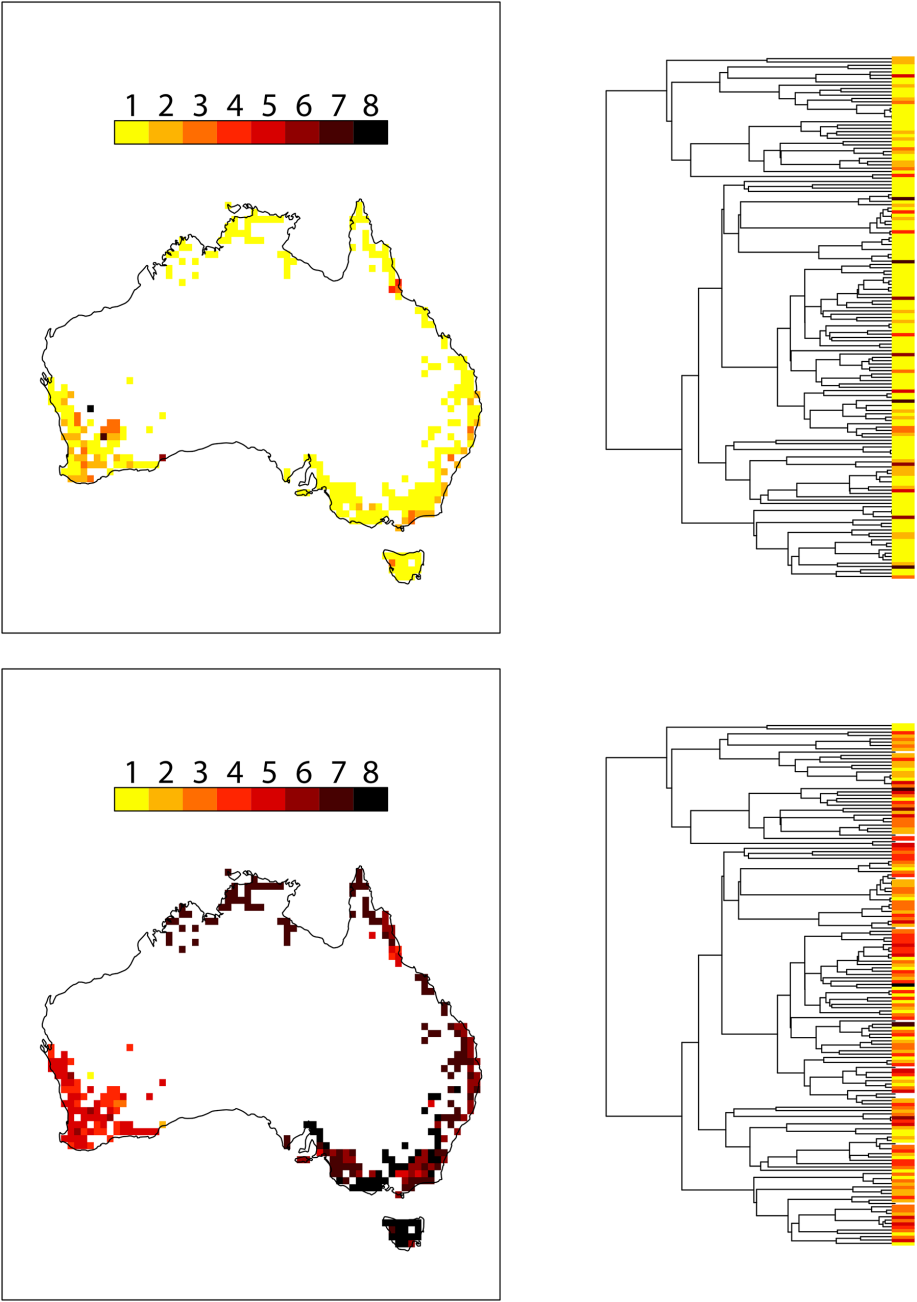
Spatial and phylogenetic patterns in the distribution of threat status and geographic range size. Spatial patterns of threat status (upper panels) and range size (lower panels) are presented as the mean value across species within each 100km^2^ grid cell, and phylogenetic patterns are presented as species-specific values. Range size is log-transformed and rescaled to the range 0–8 to give units consistent with threat status.

The spatial and phylogenetic distributions of the two response variables are presented graphically in Fig 1. Mean threat status per grid cell is low throughout most of the continental distribution of *Banksia*, but there are a few “hotspots” of elevated mean threat status, in several places along the east coast and throughout the southwest of Australia. Phylogenetic distribution of threat status reveals little phylogenetic pattern, with highly-threatened species distributed throughout the tree. The spatial pattern of mean range size per grid cell shows a clear difference between the southwest (with many narrowly-distributed species) and eastern/northern Australia, where many species are widely-distributed, but within each region shows little spatial pattern. Range size values are also scattered on the phylogeny, although the distribution of range sizes appears more clustered than threat status. These visual patterns are confirmed by statistical tests, that reveal very low levels of phylogenetic signal in threat status (Pagel’s λ = 0, *p*(λ = 0) = 1), but significant phylogenetic signal in range size (Pagel’s λ = 0.34, *p*(λ = 0) < 0.0001), and low levels of spatial signal in both mean threat status (Moran’s I = -0.003, *p* = 0.53) and mean range size (Moran’s I = -0.008, *p* = 0.75).

Univariate tests of each predictor against the two response variables, including the spatial and phylogenetic variance-covariance matrices, are summarized in Table 1 and Table 2. For threat status models, two predictors were significant: length of flowering period (negative association) and soil depth (negative), both of which remained significant in a multiple regression model and were used as the predictor set for further model comparisons. For range size models, six predictors were significant: maximum height, length of flowering period, resprouting fire response, habitat structural complexity, mean annual precipitation, and % remaining vegetation cover. When these six variables were fitted simultaneously in a multiple regression, precipitation dropped out of the model with *p* > 0.05. The remaining five were used as the predictor set for further model comparisons.

**Table 1:** Threat status models. Results are shown for univariate tests of each predictor against threat status, with parameter estimates independent of phylogenetic and spatial effects.

Predictor	Intercept	Slope estimate	Slope s.e.	*p*
Adult height	0.73	-0.03	0.03	0.16
Length of flowering period	1.38	-0.17	0.05	< 0.001
Seed size	0.88	-0.01	0.013	0.153
Resprouter	0.76	-0.09	0.4	0.359
Habitat complexity	0.76	-0.05	0.09	0.312
Precipitation	0.7	0.001	0.005	0.441
Temperature	1.59	-0.05	0.06	0.21
Soil pH	1.32	-0.12	0.32	0.348
Soil depth	3.21	-2.59	1.05	0.007
% Vegetation cover	0.87	-0.73	0.69	0.145

**Table 2:** Range size models. Results are shown for univariate tests of each predictor against log(range size), with parameter estimates independent of phylogenetic and spatial effects.

Predictor	Intercept	Slope estimate	Slope *s*.*e*.	*P*
Adult height	2.5	0.12	0.02	< 0.001
Length of flowering period	1.65	0.2	0.04	< 0.001
Seed size	2.34	0.008	0.01	0.216
Resprouter	2.37	0.36	0.18	0.029
Habitat complexity	1.71	0.35	0.07	< 0.001
Precipitation	1.71	0.02	0.004	< 0.001
Temperature	3.27	-0.05	0.04	0.155
Soil pH	2.7	0.05	0.24	0.412
Soil depth	1.2	1.33	0.83	0.056
% Vegetation cover	1.91	2.63	0.48	< 0.001

Results of model comparisons are shown in Table 3 and Table 4. For threat status models (Table 3), the fit of a non-phylogenetic, non-spatial model that included only the predictor set provided the best fit to the data (ΔAIC = 0), although the three models that added spatial and/or phylogenetic effects to the predictors were only slightly worse (ΔAIC = 1.49–3.12). The model that excluded the predictors and modelled threat status only as a function of spatial and phylogenetic effects was a substantially worse fit (ΔAIC = 16.56). Results were similar for range size models (Table 4). The best fitting model included the predictors only (ΔAIC = 0), but the models including predictors + space, predictors + phylogeny, and predictors + space + phylogeny were only marginally worse (ΔAIC = 0.95–2.94). Again, the model that omitted the predictors and modelled range size as a function of phylogenetic and spatial effects only was substantially worse (ΔAIC = 62.14).

**Table 3:** Model comparisons for threat status models.

Model	AIC	ΔAIC	*R*^2^	Variance component parameters		
				Independent (γ)	Phylogenetic (λ’)	Spatial (φ)
Predictors only	249.11	0	0.097	-	-	-
Phylogeny + space only	265.67	16.56	0	0.995	0.003	0.001
Predictors + phylogeny	250.6	1.49	0.095	0.995	0.003	-
Predictors + space	250.9	1.79	0.096	0.995	-	0.005
Predictors + phylogeny + space	252.23	3.12	0.097	0.989	0.004	0.006

**Table 4:** Model comparisons for range size models.

Model	AIC	ΔAIC	*R*^2^	Variance component parameters		
				Independent (γ)	Phylogenetic (λ’)	Spatial (φ)
Predictors only	121.77	0	0.342	-	-	-
Phylogeny + space only	183.91	62.14	0	0.995	0	0.005
Predictors + phylogeny	123.77	2	0.341	1	0	-
Predictors + space	122.72	0.95	0.16	0.991	-	0.009
Predictors + phylogeny + space	124.71	2.94	0.163	0.991	0	0.009

The total explanatory power of the models was low for threat status (*R*^2^ = 0–0.097), but considerably higher for range size (*R*^2^ = 0–0.342). In the threat status models, nearly all of the variation was independent of space and phylogeny (*γ* = 0.989–0.995), with very small marginal contributions of space (*φ* = 0.001–0.006) and phylogeny (*λ’* = 0.003–0.004). In the range size models also, nearly all of the variation was independent of space and phylogeny (*γ* = 0.991–0.995), with very small space (*φ* = 0.005–0.009) and zero phylogeny (*λ’* = 0) components.

## Discussion

Since the pioneering study of Purvis et al.[17] it has become standard practice for comparative studies of extinction risk to use methods that minimize or remove possible effects of phylogenetic non-independence. During the same period the importance of spatial autocorrelation in geographic data has also become more widely appreciated by ecologists and biogeographers[29]. Because the three basic elements of comparative extinction risk models (threatening processes, biological traits, environmental variables) are often expected to be spatially autocorrelated, spatial non-independence may be just as influential as phylogenetic non-independence in extinction risk models. However, it is too early to judge whether this is supported empirically, because to our knowledge only two studies so far have analyzed patterns of extinction risk across species using methods that incorporate the independent effects of space and phylogeny[13,14]. Both studies were global-scale analyses of mammals, and although they used different analytical approaches, both found substantial spatial and phylogenetic contributions to extinction risk variation in some mammal taxa.

In contrast, our results for *Banksia* show that neither phylogeny nor space account for much of the variation in threat status or range size, with nearly all of the variance explained by effects independent of space and phylogeny (i.e., the *γ* parameter). This is the case both when biological and external predictors are included in the models, and when phylogeny and space alone are included, without the predictors. The very low phylogenetic and spatial variance components (*λ’*and *φ*) may indicate one or more of the following: (1) the significant predictors of extinction risk in our models show little phylogenetic or spatial signal; (2) the predictors themselves explain only a small component of the variance in extinction risk; (3) much of the variance in extinction risk of *Banksia* is due to factors not included in our models, that show very little phylogenetic or spatial signal. All three of these explanations are probably true to some degree. For threat status, the only significant biological correlate is length of flowering period, which has very low phylogenetic signal (λ = 0.062). For range size, on the other hand, one of the significant predictors is plant height, which does show substantial phylogenetic signal (λ = 0.61), but the contribution of plant height to range size variance is comparatively low (*R*^2^ = 0.15). A number of spatial variables are also associated with threat status and range size, but again, both the strength of their spatial signal and their contribution to the variance is comparatively low, leading to low estimates for the spatial effect in threat status and range size models.

The low overall contribution of biological and environmental predictors to the variance in threat status is also consistent with the idea that extinction risk in some plant groups may be a legacy of the speciation process, and therefore largely independent of recent anthropogenic impacts. In the flora of South Africa’s Cape Province, another Mediterranean-climate hotspot, Davies *et al*. [30]found that highest proportions of threatened species were found in young and rapidly-diversifying genera, which they attributed to a prevailing peripatric speciation process that leaves many species with very restricted distributions, and hence a higher threat status. In *Banksia*, although speciation rates are not unusually high[20], many species in the SWBP are known only from very restricted distributions, which probably reflects a long history of allopatric speciation and edaphic specialization[31,32]. In this way, the natural rarity of many SWBP *Banksia* species has put them at immediate risk in an environment changing rapidly under recent human impact.

Notwithstanding the comparatively low explanatory power, the sets of significant predictors in our models confirm that *Banksia* fits the general model that extinction risk is influenced by a combination of biological traits, environmental features and external threatening processes. The sole biological predictor of threat status was length of flowering period. This can be understood as a demographic indicator: species that flower for a longer period each year have a longer recruitment period and can achieve higher abundances[9], and this can result in species with long flowering periods becoming more prevalent in a community than expected by chance [33]. Flowering period was also correlated with range size, perhaps via the well-known positive association between abundance and geographic range size[34,35]. Several previous studies support a link between longer flowering periods and measures of range size or rarity in plant groups as divergent as Australian Eucalypts [1], Finnish vascular plants[36], and British meadow grassland species [9], suggesting that the pattern may be a very general one. On the other hand, it is also possible that the enhanced detectability of plants in flower leads to a sampling bias, so that the abundance of species with shorter flowering periods is underestimated [9], potentially leading to a misassignment of threat status or an underestimate of range size [25]. This could in fact be an important consideration for *Banksia* growing in dense heathlands, where many small species become far more conspicuous and easy to identify when their large, showy inflorescences are in bloom. A similar sampling artefact may explain the positive association between plant height and range size, if abundances of small plants are likely to be underestimated.

In some previous studies, the probability of extinction of plant species has been linked with pollination mode, although in conflicting ways: in Singapore, the most extinction-prone angiosperms were those dependent on mammal pollinators [7], while other studies have found highest extinction risk in species pollinated by insects [1,37,8]. We have not included pollination mode in our analyses because of a lack of detailed data, but we suspect that pollination mode is unlikely to have a major influence on extinction risk in *Banksia*. Most *Banksia* species are bird-pollinated, but many are also pollinated by small mammals, and the comparatively low pollinator specificity of *Banksia* species means that seed set rates are rarely limited by the availability of pollinators [6].Our results also indicate that the most direct measure of threat to plant species (habitat loss) is a significant predictor of extinction risk in *Banksia*. Therefore, although the particular responses of different species to threatening processes is context-dependent, varying with biological traits and environmental features, the direct, independent effect of habitat loss is still evident. This should not be surprising given that habitat loss has not only reduced population and geographic range sizes, but may also have led to non-optimal fire regimes and greater susceptibility to weeds or diseases [6]. Furthermore, small, fragmented populations of some *Banksia* species have been shown to produce fewer seeds per plant compared to larger populations [6].This confirms the conclusion of Murray *et al*. [10] that comparative extinction risk models should always incorporate variables that capture direct threatening processes, as well as biological traits and environmental variables.

## Supporting Information

S1 Table Biological and environmental data for Banksia species. (XLSX)

S2 Table Geographic centroid coordinates for Banksia species. (CSV)

S1 Text R code for the analyses. (R)
